# Sperm DNA Fragmentation after Cryopreservation and Sperm Selection Has No Implications for Clinical Pregnancies and Live Births after Intrauterine Insemination with Donor Sperm

**DOI:** 10.3390/jpm13121668

**Published:** 2023-11-28

**Authors:** Alessa Sugihara, Usha Punjabi, Tiziana Chimienti, Ilse Goovaerts, Kris Peeters, Jason Bouziotis, Diane De Neubourg

**Affiliations:** 1 Centre of Reproductive Medicine, University Hospital of Antwerp, 2650 Edegem, Belgium; 2 Faculty of Medicine and Health Sciences, University of Antwerp—Campus Drie Eiken, 2610 Wilrijk, Belgium; 3 Centre of Reproductive Medicine, Algemeen Ziekenhuis KLINA, 2930 Brasschaat, Belgium; 4 Clinical Trial Center, University Hospital of Antwerp, 2650 Edegem, Belgium

**Keywords:** intrauterine insemination, donor sperm, heterologous insemination, sperm DNA fragmentation, sperm quality, cryopreservation, density gradient centrifugation, fertility potential, reproduction rate

## Abstract

Intrauterine insemination with donor sperm (IUI-D) requires multiple in vitro manipulations such as sperm selection and cryopreservation during which spermatozoa may be exposed to oxidative stress (OS) and other insults that may produce potential damage including sperm DNA fragmentation (SDF). High levels of SDF, referring to damage or breaks in the genetic material of sperm cells, are linked to an increased risk of reproductive failure. This retrospective, observational study set out to evaluate whether SDF assessment could predict clinical outcome in an IUI-D program, where sperm donors are selected on strict conventional semen parameters. A total of 18 donors and 106 recipients were matched for IUI-D. Out of 429 cycles, 100 (23.3%) resulted in clinical pregnancy. We counted 78 live births (18.2% of cycles), while 20 pregnancies ended in miscarriage (4.7% of cycles), 1 in extra-uterine pregnancy and 1 in stillbirth. Female age significantly influenced clinical pregnancy and miscarriage rates. SDF increased after cryopreservation (26.3 ± 14.5%; *p* < 0.001) and more so after post-thaw density gradient (34.9 ± 22.1%; *p* = 0.04) without affecting clinical pregnancy (OR [95% CI] 1.01 [0.99; 1.02]; *p* = 0.27), live birth (1.00 [0.99; 1.02]; *p* = 0.72) and miscarriage rates (1.02 [1.00; 1.05]; *p* = 0.08). The implications of our findings extend to a better selection of sperm donors and a better sperm preparation technique tailored to the donor semen’s properties in order to maximize the chances of a favorable treatment outcome.

## 1. Introduction

Intrauterine insemination (IUI) is a non-invasive, first-line treatment of assisted reproduction, which is performed either with husband/partner sperm (IUI-H) in cervical, idiopathic or mild/moderate male infertility cases [1], either with heterologous/donor (IUI-D) sperm most often for same sex female couples or single women. Medically assisted reproduction, encompassing both IUI and in vitro fertilization/intracytoplasmic sperm injection (IVF/ICSI), entails multiple in vitro manipulations such as sperm selection, cryopreservation and other incubation procedures, during which spermatozoa may be exposed to oxidative stress (OS) and other insults that may produce potential damage including sperm DNA fragmentation (SDF), membrane modifications, mitochondrial function and morphology alterations [2].

For both IUI-H and IUI-D, sperm selection is inevitable to enable selection of highly motile, morphologically normal spermatozoa for insemination. Various techniques are implemented, with density gradient centrifugation (DGC) being the most common method. While this technique can recover the highest motile percentage of normal spermatozoa in both normal and subnormal semen samples, studies have reported an increase in sperm DNA damage after density gradient centrifugation with metal contamination and intrinsic sperm characteristics probably being the culprits here [3,4].

Donor sperm used for IUI-D undergoes an additional manipulation, i.e., cryopreservation. Although sperm cryopreservation facilitates the storage of donor semen for IUI-D while infectious diseases screening can be completed and confirmed negative, viability and motility remain vulnerable parameters during the freeze-thaw process with the possibility of OS inducing additional SDF due to differences in cryotolerance and implemented cryopreservation methods [5].

A certain degree of DNA fragmentation is inherent to the process of chromatin compaction, but, high levels of SDF have been linked to lower fertilizing potential of the sperm [6], lower clinical pregnancy numbers [7] and higher risk of miscarriage [8]. Moreover, a significant number of subfertile men have abnormal sperm DNA integrity despite normal semen parameters [9,10,11]. A systematic review concluded that sperm DNA damage was associated with lower IUI-H pregnancy rates [12]. Later, an updated review found that low SDF was associated with higher chances of clinical pregnancy after IUI (relative risk = 3.15) [13]. In the ID-trial, a prospective cohort IUI-H study investigating the relationship between sperm DNA fragmentation and intra-uterine insemination outcome in couples with unexplained or mild male infertility, we found an inverse relationship between SDF in the ejaculate of the diagnostic semen sample and clinical pregnancy [14].

Embarking on these findings we aimed to evaluate whether SDF assessment could predict clinical outcome in an IUI-D program, where sperm donors are selected on strict conventional semen parameters.

## 2. Materials and Methods

### 2.1. Study Design and Participants

This was a retrospective, observational study and the project was approved by the Ethical Commission of the Antwerp University Hospital and the University of Antwerp on 26 June 2017, ref. no: 17/24/285 (Belgian registration no: B300201732872). Between March 2015 and March 2019, screening of potential sperm donors was conducted in accordance with the Belgian Tissue and Cell Bank regulations [15] following a three-step plan: semen assessment, medical assessment and serological blood tests. According to the Belgian legislation (2007), a sperm donor was matched with a maximum of 6 women/couples whereas more than one offspring by that donor in one couple was accepted. To clarify, a donor can be matched with more than 6 different women in case of same sex female couples where both women can be matched to the same donor or in case a woman did not come pregnant after sperm donation and stopped treatment resulting in a new matching possibility for that donor.

**Figure 1:**
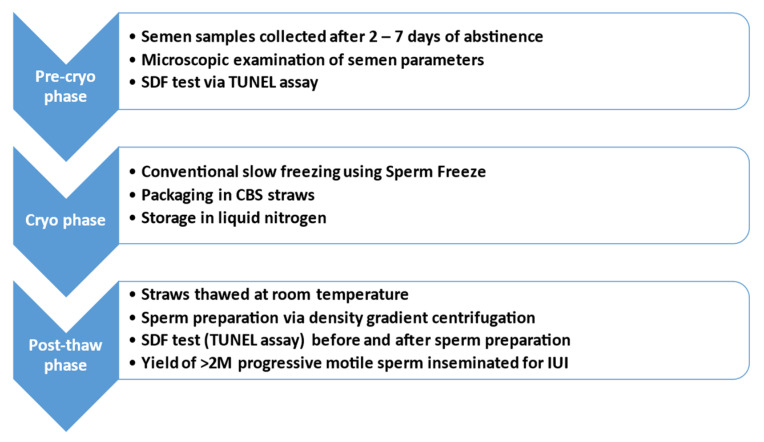
Different phases of the study. SDF = sperm DNA fragmentation; CBS = CBS^TM^ High security sperm straw; M = million.

IUI reimbursement (Belgium) considers the female age (until the 43rd birthday) but not the male age. The different phases of the study are depicted in Figure 1.

### 2.2. Ovarian Stimulation/Cycle Monitoring

IUI was predominantly performed in spontaneous cycles, ovarian stimulation either with clomiphene citrate or low-dose gonadotrophins occurred in 18% of all cycles. When 1 or 2 dominant follicles were present, IUI was planned 34 to 38 h after hCG-trigger (250 µg hCG or 5000 IU (Ovitrelle^®^, Merck nv, Overijse, Belgium or Pregnyl^®^ MSD Belgium, Brussels, Belgium) or 24–28 h after detection of spontaneous LH surge. When three or more dominant follicles were present, a reduction to 1 or 2 follicles was performed or the cycle was cancelled according to the patients’ preference.

### 2.3. Semen Analysis

Sperm donors were instructed to maintain 2–7 days of sexual abstinence. All semen samples were collected at the laboratory and any ejaculate fraction missing was reported. Samples were weighed and analysis was initiated within 60 min after ejaculation according to WHO 2010 recommendations [16]—including sperm concentration using improved Neubauer hemocytometer (Marienfeld GmbH, Lauda-Königshofen, Germany) combined with a positive displacement pipette (Microman, Gilson Inc, Middleton, WI, USA); sperm motility included progressive and total motile sperms and sperm morphology adapting the modified papanicolaou stain (Sigma-Aldrich Inc., St. Louis, MO, USA).

### 2.4. Semen Cryopreservation

Semen samples were frozen after liquefaction maximum 1 h after production. The ejaculate was slowly diluted 1:1 with SpermFreeze Solution (Vitrolife, Gothenburg, Sweden). After 10 min, 0.5 mL CBS^TM^ high security sperm straws (Cryo Bio System, L’Aigle, France) were filled and sealed. The straws were frozen with an automatic freezing protocol (CL8800i, CryoLogic, Blackburn, VIC, Australia) starting at 24 °C, followed by −5 °C/min until 4 °C. After holding for 1 min on 4 °C, freezing continued at a rate of −8 °C/min until −80 °C, ending in free fall. The straws were stored in liquid nitrogen vapour.

### 2.5. Sperm Thawing and Processing

The straws were thawed at room temperature and the contents treated with a two-step (40% and 80%) discontinuous density gradient using Puresperm^®^ (Nidacon, International AB, Gothenburg, Sweden) [17]. After processing the final pellet was washed, with human tubal fluid (HTF Hepes, Gynotec, Malden, The Netherlands) supplemented with albumin (Human Albumin 20%, CAF-DCF, Brussels, Belgium).

### 2.6. SDF Assessment

Terminal deoxynucleotidyl transferase-mediated deoxyuridine triphosphate nick-end labeling (TUNEL assay) was used to assess SDF as described by Mitchell et al. [18]. Briefly, the sperm cells were incubated with 2 mM dithiothreitol (DTT, Sigma-Aldrich, Overijse, Belgium) for 45 min. After washing with phosphate-buffered saline (PBS, GIBCO Life technologies, Paisley, UK) the samples were fixed in 3.7% formaldehyde (Sigma-Aldrich, Belgium) for 20 min at 4 °C. The semen analysis and SDF assay were carried out at three time-points: directly on fresh semen samples without storage, after cryopreservation and after processing with density gradient centrifugation (post-thaw). For the assay, the spermatozoa were resuspended in 500 µL of fresh permeabilization solution (100 mg Sodium citrate, 100 µL Triton X–100 in 100 mL dH2O). The positive control samples were treated with 5 µL of DNase I (Qiagen, Hilden, Germany) 1500 Kunitz Units for 30 min at room temperature. The assay was performed using the fluorescein In Situ Cell Death Detection.

Kit (Roche Diagnostics, Mannheim, Germany) using an Accuri C6 flow cytometer (BD Sciences, Erembodegem, Belgium) recorded 5000–10,000 events for each sample at a flow rate of 35 µL/min. The method has been standardized and cut-off values were defined [19,20].

### 2.7. Intrauterine Insemination

Before insemination, the motility and the concentration were assessed, and the total inseminating progressive motile count (IMC) of ≥2 × 10^6^ progressive motile spermatozoa [20] was inseminated using a soft IUI catheter (Wallace^®^ Intrauterine Insemination Catheters, Cooper Surgical, The Hague, The Netherlands) rinsed with HTF and albumin. The inseminating volume was held constant between 0.3–0.5 mL [21].

### 2.8. Treatment Outcome

A clinical pregnancy (CP) with fetal heartbeat was diagnosed by ultrasonography. A miscarriage was defined as the spontaneous loss of an intrauterine pregnancy prior to 22 completed weeks of gestational age. Live births (LB) were registered in case of a birth occurring after 22 completed weeks of gestational age with evidence of life [22].

### 2.9. Statistical Analysis

Statistical analyses were performed with Stata/SE 17.0 and R version 4.2.2. Descriptive statistics of donor’s and recipient’s data are presented overall and in the different outcome groups. The normality of the continuous variables was assessed based on graphical representations (histogram, box plot). Data are presented as mean ± standard deviation for normally distributed variables and median (interquartile range) for asymmetrical distributions. Both absolute (*n*) and relative (%) frequencies are presented for categorical data. Paired data were compared with Student’s paired t test or Wilcoxon signed-rank test, depending on the normality of the difference.

Reproduction rate (RR), defined as the number of women with a clinical pregnancy divided by the number of receptors per donor, was calculated to determine a donor’s fertility potential.

Probability of clinical pregnancy, live birth and miscarriage according to the donor’s age, motility, DNA damage, and cycle variables were analyzed using multilevel mixed-effects logistic regression. The models included one outcome with only one explanatory variable at a time. Following which every association was adjusted for the woman’s age. Random effects for each woman and for each donor have been specified to account for correlation between data from the same woman and from the same donor. These random effects were crossed as two women had been inseminated with sperm from different donors. Odds ratios (OR) with 95% confidence intervals and Wald’s test *p*-values are reported. Time-to-live birth was analyzed with mixed-effects Cox regression models with Breslow method for ties, considering the number of cycles as the time variable. We reported hazard ratios (HR) with 95% confidence intervals and Wald’s test *p*-value. Additionally, we analyzed the correlation between RR and SDF with Pearson’s correlation coefficient. Statistical significance was set at *p* value < 0.05.

## 3. Results

### 3.1. Descriptive Statistics

Descriptive statistics for donor and recipient variables are presented in Table 1. Mean female age at first cycle was 33.9 ± 4.1 years and donor age was 28.5 ± 5.6 years. Data were available for 429 cycles from 106 different women who received sperm from 18 different donors (1 or 2 donors per woman). Donors inseminated 1 to 11 different women with an average of 6 ± 2.6 women.

**Figure 2:**
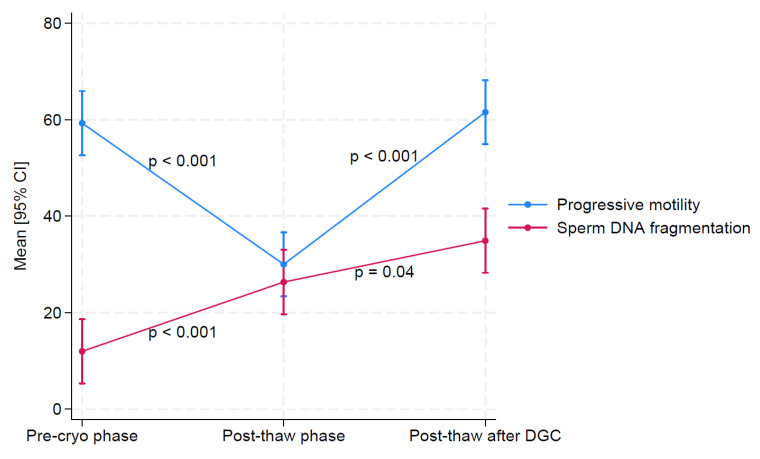
Progressive motility and SDF in the different phases of the study.

Progressive motility (59.3 ± 12.5%) decreased significantly after cryopreservation (30.0 ± 13.2%; *p* < 0.001) but increased after density gradient centrifugation (post-thaw) (61.6 ± 16.0%; *p* < 0.001). On the contrary, SDF (12.0 ± 5.9%) increased post-cryopreservation (26.3 ± 14.5%; *p* < 0.001) and further increased after sperm selection (34.9 ± 22.1%; *p* = 0.04) (Figure 2).

### 3.2. Pregnancy Outcome

Out of 429 cycles included, 100 (23.3%) resulted in clinical pregnancy. We counted 78 live births (18.2% of cycles), while 20 pregnancies ended in miscarriage (4.7% of cycles), 1 in extra-uterine pregnancy and 1 in stillbirth. There were no reports of congenital anomalies.

**Figure 3:**
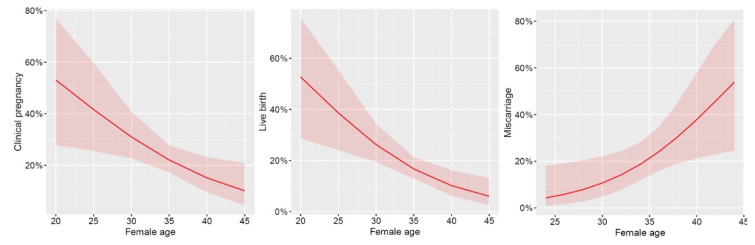
Predicted probabilities of clinical pregnancy, live birth and miscarriage according to female age. The shaded area represents the 95% confidence interval.

Using multilevel mixed-effects logistic regression (Odds ratio [95%CI]), we found that female age significantly influenced clinical pregnancy (0.911 [0.847–0.981]; *p* = 0.01), live birth (0.894 [0.834–0.959]; *p* = 0.002) and miscarriage rates (1.180 [1.033–1.347]; *p* = 0.015) (Figure 3).

Although donor selection was based on age, normal semen parameters and post-thaw survival of >50% of initial motility, in 7/18 donors SDF was above the threshold criteria of normality >13% in the neat semen [20]. CP was not statistically different (*p* > 0.05) whether SDF was ≤13% (58/261; 22.2%) or >13% (42/168; 25.0%). There was no significant female-age-adjusted effect of sperm motility and SDF pre- and post-thaw and after DGC post-thaw on clinical pregnancy (Table 2), live birth (Table 3) or miscarriages (Table 4). Concurrently, IUI performed in spontaneous or stimulated cycles (1.652 [0.786; 3.471]; *p* = 0.19), the number of straws thawed to prepare the inseminate (1.001 [0.923; 1.086]; *p* = 0.98), the IMC (0.992 [0.850; 1.156]; *p* = 0.91) and the number of cycles undertaken to achieve a pregnancy (0.979 [0.842; 1.139]; *p* = 0.79) had no significant effect on CP even after adjusting for recipient age (the OR presented here were adjusted for female age).

### 3.3. Time-to-Live Birth

**Figure 4:**
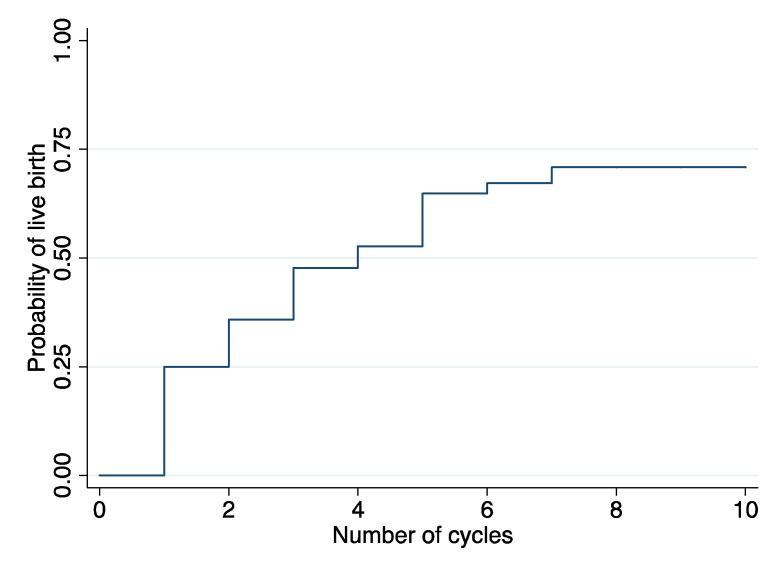
Time-to-live birth.

Median time-to-live birth was 4 cycles (Figure 4). Analyses of time-to-live birth with mixed-effects Cox models revealed no significant association with sperm progressive motility pre-cryo (0.996 [0.978–1.014]; *p* = 0.68), post-thaw (0.998 [0.980–1.017]; *p* = 0.82) and after DGC post-thaw (1.001 [0.988–1.015]; *p* = 0.86). The same trend was observed with SDF before (1.010 [0.967; 1.054]; *p* = 0.66) or after cryopreservation (1.003 [0.986; 1.021]; *p* = 0.75) and after sperm selection post-thaw (1.003 [0.992; 1.014]; *p* = 0.59), even after adjusting for recipient age (the OR presented here were not adjusted for female age).

### 3.4. Fertility Potential

Fertility potential expressed as reproduction rate (RR) was 72 ± 15%. Weak to moderate but not statistically significant associations were found between RR and SDF pre-cryo (r = −0.33; *p* = 0.18); SDF post-thaw (r = −0.28; *p* = 0.27) and SDF post-thaw after DGC (r = −0.33; *p* = 0.19).

## 4. Discussion

High levels of sperm DNA fragmentation, referring to damage or breaks in the genetic material of sperm cells, are linked to an increased risk of reproductive failure. As such, selecting and identifying sperm for assisted reproductive techniques (ART-IUI/IVF/ICSI) with low levels of fragmentation is thought to optimize patient’s treatment and improve the chances of a successful pregnancy. In light of the ongoing rise in the utilization of donor sperm [23], this study sought to analyze the IUI-D outcome, with regard to the SDF levels both before and after sperm selection and cryopreservation.

Firstly, our study did not find a significant relationship between SDF and clinical pregnancy nor live birth in IUI-D treatments. These findings stand in contrast to Lu et al. who found that IUI donors with a higher pregnancy rate had a significantly lower sperm DNA fragmentation index (DFI) and concluded that DFI might be a good predictor for IUI-D [24]. Unfortunately, there was no disclosure on the definition of pregnancy nor information pertaining to miscarriage or live birth. Furthermore, Hu et al. found that an increased sperm DNA Fragmentation index was linked to a lower RR in the setting of intracervical insemination (without sperm processing) [25]. Our finding is also contrary to results from IUI-H studies which suggest that increased SDF is related to detrimental IUI outcome [13].

Surprisingly, there was also no significant difference for SDF and miscarriage although the analysis almost seems to suggest an association between low SDF post-thaw after DGC and miscarriage (*p* = 0.08). Perhaps the lack of a statistically significant association between SDF and miscarriage in our study, which diverges from the current literature [26], can be partly explained by the relatively small number of miscarriages (*n* = 20).

Moreover, our results indicate a clinical pregnancy rate of 23.3% of cycles which is higher than the overall international reported pregnancy rates of IUI-D ranging between 6.4% and 18.8% [27,28] and higher than the average reported by the latest Belgian Register of Assisted Procreation (BELRAP, 2020) [29]. The median time to pregnancy of 4 IUI-D cycles appears to be in line with previous observations where pregnancy rates seem to decline between the 6th and 10th IUI-D cycle [28,30,31], although directly comparable data are lacking.

The mean reproduction rate of 72% reflects the donor’s high fertility potential and is in line with our previous findings [32] but significantly higher than the RR of 26.8% reported by Hu et al. [25]. Though this discrepancy may reflect the study center’s qualitative and stringent donor screening process, we must also stress the important difference in insemination technique, namely intrauterine versus intracervical.

Most sperm donors, except for 7, had low levels of pre-cryo SDF. However, these levels increased after cryopreservation/thawing and continued to rise after sperm selection with DGC. Furthermore, although sperm used for IUI-D undergoes an additional manipulation (cryopreservation), results obtained with IUI-H are lower than those obtained with IUI-D with international registries reporting IUI delivery rates of 8.9% and 11.7%, respectively [33,34]. Perhaps these findings may be explained by the ability of the oocyte of an otherwise not proven subfertile woman (single or same sex female couple) to overcome high levels of SDF whereas women undergoing IUI-H represent a more heterogenous group with infertility [35].

Additionally, our results confirm the broadly studied, inverse relationship between female age and live birth rate [27,36]. Remarkably, donor age was not associated with pregnancy, live birth or miscarriage rates. These results are in line with the findings of a large retrospective study which did not find an unfavorable effect of advancing sperm donor age [37]. However, we acknowledge our relatively young donor population of <45 years might not reveal an association. Our results may therefore not be extrapolated as such to donors >45 years, especially given the possible association between advanced paternal age and long-term health implications for the offspring including autism, schizophrenia, bipolar disorders and pediatric leukemia [38].

Sperm cryopreservation is an important technique of fertility management in ART both in terms of autologous and heterologous sperm use. Nonetheless there are a vast number of factors that could potentially influence the outcome of cryopreservation and cause a significant effect on the genetic integrity [39]. Plasma membrane functionality, motility and overall sperm viability post-thaw typically decreases in contrast to the pre-freeze state [40,41]. In addition, cryopreservation changes mitochondrial membrane properties and increases the production of reactive oxygen species (ROS), subsequently resulting in the oxidation of DNA which in turn can produce high frequencies of DNA breaks [42]. Although the available literature shows conflicting results [43], there are many indicators of an increase in SDF after freeze/thaw both in subfertile and fertile men [44,45,46]. Our results show a significant increase in SDF after cryopreservation and thus confirms this trend in a donor population. Perhaps this warrants an exploration of other techniques for cryopreservation which induce less sperm DNA damage such as vitrification [47], although its applicability may be restricted owing to its labor-intensive nature and higher costs.

Lastly in vitro manipulation involves the separation of cells from the seminal plasma and the removal of immature sperm and leukocytes which are the main sources of intracellular ROS [10,48,49]. However, results are controversial as to the effect of DGC on SDF with reports that DGC does not induce any significant increase in sperm DNA damage [50] or even decreases SDF [51] while conflicting studies report an increase in SDF [4] after DGC. Our previous observations have in fact shown that SDF can either increase, decrease or remain status quo after DGC depending on the patient category [52].

Certain limitations warrant consideration in the interpretation of our results. Firstly, the data was sampled from a relatively small donor population. Secondly, the results pertain to donors or men with high normal semen parameters and thus limits the generalizability to subfertile men with normal or subnormal semen parameters. Additionally, adjustments for potential compounding factors, such as smoking status and BMI of both donor and recipient women were not possible. Lastly, SDF analysis through TUNEL with flow cytometry requires specialized infrastructure and remains labor intensive resulting in a higher lab cost. As such, its routine performance is limited as long as the evidence remains conflicting. The included patients were not charged any additional costs for the purpose of the study.

## 5. Conclusions

We did not find a significant relationship between SDF and live birth for patients undergoing IUI-D. Despite the aforementioned limitations, we believe our study lays the groundwork for continued exploration of SDF testing in IUI-D. Moving forward, the implications of our findings extend to a better selection of sperm donors and a better sperm preparation technique tailored to the donor semen’s properties in order to maximize the chances of a favorable treatment outcome and even more so, healthy offspring. Future studies should also explore the effect of SDF on the long-term health of the donor’s offspring. Perhaps other sperm freezing and selection techniques can be studied offering comparable high live births after IUI-D while decreasing SDF, ideally in a randomized controlled setting.

## Figures and Tables

**Table 1:** Descriptive statistics.

Variables	Value
**Donor variables (*n* = 18):** Age (years) Abstinence (days) Semen volume (mL) Sperm concentration (M/mL) Progressive sperm motility (%) Total sperm motility (%) Sperm morphology (%) SDF (%)	28.5 ± 5.6 [20.2–40.1] 3.0 ± 0.6 [2.0–5.0] 4.2 ± 2.0 [2.1–10.1] 80.0 ± 43.9 [20.8–184.6] 59.3 ± 12.5 [26.0–85.0] 67.1 ± 12.8 [30.0–86.0] 8.9 ± 2.6 [5.0–14.0] 12.0 ± 5.9 [4.3–22.1]
**Recipient variables (*n* = 106):** Age (years) Spontaneous cycle IUI (%) Stimulated cycle IUI (%) Number of straws thawed Inseminating progressive motile count (M) Number of IUI cycles undertaken	33.9 ± 4.1 [24.6–41.9] 352/429 (82.1%) 77/429 (17.9%) 6.5 ± 3.5 [3–23] 3.2 ± 1.7 [0.5–10.5] 4.0 ± 2.3 [1–10]

Data are presented as Mean ± SD [full range] or frequencies; SDF = sperm DNA fragmentation; M/mL = million/milliliter.

**Table 2:** Influence of age, sperm motility and SDF parameters on clinical pregnancy rates.

	No Clinical Pregnancy (*n* = 329)	Clinical Pregnancy (*n* = 100)	OR [95% CI]	*p*-Value	OR [95% CI] Adj. for Woman’s Age	*p*-Value
Donor age (years)	28.8 ± 5.4	29.5 ± 6.1	1.02 [0.97; 1.08]	0.39	1.02 [0.96; 1.07]	0.56
Recipient age (years)	35.1 ± 4.0	33.8 ± 4.1	0.91 [0.85; 0.98]	0.01		
Ovarian stimulation	57 (17.3)	20 (20.0)	1.47 [0.70; 3.06]	0.31	1.65 [0.79; 3.47]	0.19
Pre-cryo progressive motility (%)	59.5 ± 12.1	59.1 ± 12.4	1.00 [0.97; 1.02]	0.73	1.00 [0.98; 1.02]	0.93
Pre-cryo total motility (%)	68.0 ± 12.8	67.5 ± 13.0	1.00 [0.97; 1.02]	0.68	1.00 [0.98; 1.02]	0.90
Post-thaw progressive motility (%)	29.6 ± 11.7	29.3 ± 13.3	1.00 [0.98; 1.02]	0.95	1.00 [0.98; 1.03]	0.88
Post-thaw total motility (%)	33.8 ± 12.3	34.3 ± 14.7	1.00 [0.98; 1.03]	0.71	1.01 [0.98; 1.03]	0.58
Post-thaw after DGC progressive motility (%)	62 [52; 72]	62 [52; 72]	1.00 [0.99; 1.02]	0.78	1.00 [0.99; 1.02]	0.73
Post-thaw after DGC total motility (%)	71 [57; 77]	70 [57; 77]	1.00 [0.99; 1.02]	0.78	1.00 [0.99; 1.02]	0.67
Pre-cryo SDF (%)	12.1 ± 5.2	12.3 ± 5.3	1.01 [0.96; 1.07]	0.66	1.02 [0.97; 1.08]	0.46
Post-thaw SDF (%)	26.1 ± 12.8	27.2 ± 12.4	1.01 [0.98; 1.03]	0.53	1.01 [0.98; 1.03]	0.62
Post-thaw SDF after DGC (%)	30.9 [13.9; 46.6]	38 [13.9; 62.8]	1.01 [0.99; 1.02]	0.27	1.01 [0.99; 1.02]	0.30

Data are presented as Mean ± SD; Median [IQR]; SDF = sperm DNA fragmentation; DGC = density gradient centrifugation; OR = odds ratio; CI = confidence interval.

**Table 3:** Influence of age, sperm motility and SDF parameters on live birth rates.

	No Live Birth (*n* = 351)	Live Birth (*n* = 78)	OR [95% CI]	*p*-Value	OR [95% CI] Adj. for Woman’s Age	*p*-Value
Donor age (years)	28.9 ± 5.5	29.3 ± 6.0	1.01 [0.956; 1.06]	0.71	1.00 [0.95; 1.05]	0.99
Recipient age (years)	35.1 ± 4.0	33.3 ± 3.9	0.89 [0.83; 0.96]	0.002		
Ovarian stimulation	62 (17.7)	15 (19.3)	1.34 [0.62; 2.93]	0.46	1.51 [0.70; 3.26]	0.29
Pre-cryo progressive motility (%)	59.5 ± 12.0	58.6 ± 13.1	0.99 [0.967; 1.02]	0.53	1.00 [0.97; 1.02]	0.74
Pre-cryo total motility (%)	68.1 ± 12.6	67.0 ± 13.8	0.99 [0.97; 1.02]	0.52	1.00 [0.98; 1.02]	0.75
Post-thaw progressive motility (%)	29.5 ± 11.8	29.3 ± 13.0	1.00 [0.97; 1.02]	0.85	1.00 [0.98; 1.02]	0.91
Post-thaw total motility (%)	33.8 ± 12.5	34.3 ± 14.4	1.00 [0.98; 1.03]	0.80	1.01 [0.99; 1.03]	0.61
Post-thaw after DGC progressive motility (%)	62 [52; 72]	62 [52; 73]	1.00 [0.99; 1.02]	0.73	1.00 [0.99; 1.02]	0.68
Post-thaw after DGC total motility (%)	71 [57; 77]	71 [57; 77]	1.00 [0.99; 1.02]	0.73	1.00 [0.99; 1.02]	0.62
Pre-cryo SDF (%)	12.1 ± 5.2	12.2 ± 5.5	1.01 [0.95; 1.07]	0.80	1.02 [0.96; 1.07]	0.53
Post-thaw SDF (%)	23.6 ± 12.8	26.6 ± 12.3	1.00 [0.98; 1.03]	0.87	1.00 [0.98; 1.02]	0.98
Post-thaw SDF after DGC (%)	31.8 [13.9; 54.8]	35.1 [13.9; 54.8]	1.00 [0.99; 1.02]	0.72	1.00 [0.99; 1.02]	0.81

Data are presented as Mean ± SD; Median [IQR]; SDF = sperm DNA fragmentation; DGC = density gradient centrifugation; OR = odds ratio; CI = confidence interval.

**Table 4:** Influence of age, sperm motility and SDF parameters on miscarriage rates.

	No Miscarriage (*n* = 80)	Miscarriage (*n* = 20)	OR [95% CI]	*p*-Value	OR [95% CI] Adj. for Woman’s Age	*p*-Value
Donor age (years)	29.2 ± 5.95	30.5 ± 6.7	1.04 [0.96; 1.12]	0.40	1.05 [0.96; 1.14]	0.30
Recipient age (years)	33.3 ± 3.9	35.8 ± 4.1	1.18 [1.03; 1.35]	**0.02**		
Ovarian stimulation	17 (21.3)	3 (15.0)	0.65 [0.17; 2.50]	0.53	0.50 [0.12; 2.04]	0.33
Pre-cryo progressive motility (%)	58.5 ± 13.0	61.7 ± 9.4	1.02 [0.98; 1.07]	0.30	1.02 [0.97; 1.07]	0.47
Pre-cryo total motility (%)	66.8 ± 13.7	70.3 ± 9.4	1.03 [0.98; 1.07]	0.28	1.02 [0.97; 1.07]	0.42
Post-thaw progressive motility (%)	29.3 ± 13.1	29.4 ± 14.2	1.00 [0.96; 1.04]	0.98	1.00 [0.96; 1.04]	0.85
Post-thaw total motility (%)	34.4 ± 14.6	33.9 ± 15.6	1.00 [0.97; 1.03]	0.89	0.99 [0.96; 1.03]	0.79
Post-thaw after DGC progressive motility (%)	62 [52; 73]	58.5 [52; 70]	0.99 [0.97; 1.02]	0.66	0.99 [0.96; 1.02]	0.62
Post-thaw after DGC total motility (%)	70 [57; 77]	70 [57; 75]	0.99 [0.97; 1.02]	0.71	0.99 [0.97; 1.02]	0.60
Pre-cryo SDF (%)	12.2 ± 5.5	12.8 ± 4.3	1.02 [0.93; 1.12]	0.64	1.00 [0.91; 1.12]	0.98
Post-thaw SDF (%)	26.4 ± 12.4	30.5 ± 11.9	1.03 [0.99; 1.07]	0.19	1.03 [0.99; 1.07]	0.17
Post-thaw SDF after DGC (%)	35.1 [13.9; 54.8]	43.2 [31.3; 63]	1.02 [1.00; 1.05]	0.08	1.02 [1.00; 1.05]	0.09

Data are presented as Mean ± SD; Median [IQR]; SDF = sperm DNA fragmentation; DGC = density gradient centrifugation; OR = odds ratio; CI = confidence interval.

## Data Availability

The data presented in this study are available from the corresponding author upon reasonable request.
